# Electrochemical Choline Sensing: Biological Context, Electron Transfer Pathways and Practical Design Strategies

**DOI:** 10.3390/biom16010023

**Published:** 2025-12-23

**Authors:** Angel A. J. Torriero, Sarah M. Thiak, Ashwin K. V. Mruthunjaya

**Affiliations:** School of Life and Environmental Sciences, Faculty of Science, Engineering & Built Environment, Deakin University, Burwood, VIC 3125, Australia

**Keywords:** choline sensing, electrochemical sensor, mediated electron transfer, direct electron transfer, complex biofluids

## Abstract

Choline is a central metabolite that connects membrane turnover, neurotransmission, and one-carbon metabolism, and its reliable measurement across diverse biological matrices remains a significant analytical challenge. This review brings together biological context, electrochemical mechanisms, and device engineering to define realistic performance targets for choline sensors in blood, cerebrospinal fluid, extracellular space, and milk. We examine enzymatic sensor architectures ranging from peroxide-based detection to mediated electron transfer via ferrocene derivatives, quinones, and osmium redox polymers and assess how applied potential, oxygen availability, and film structure shape electron-transfer pathways. Evidence for direct electron transfer with choline oxidase is critically evaluated, with emphasis on the essential controls needed to distinguish true flavin-based communication from peroxide-related artefacts. We also examine bienzymatic formats that allow operation at low or negative bias and discuss strategies for matrix-matched validation, selectivity, drift control, and resistance to fouling. To support reliable translation, we outline reporting standards that include matrix-specific concentration ranges, reference electrode notation, mediator characteristics, selectivity panels, and access to raw electrochemical traces. By connecting biological requirements to mechanistic pathways and practical design considerations, this review provides a coherent framework for developing choline sensors that deliver stable, reproducible performance in real samples.

## 1. Introduction

Choline plays a central role in human physiology and neurochemistry, serving as the precursor of acetylcholine (ACh), an essential building block for phospholipids such as phosphatidylcholine and sphingomyelin, and as a key contributor to methyl-group metabolism and lipoprotein assembly [1,2,3]. Choline deficiency produces measurable organ dysfunction that reverses upon repletion, including hepatic steatosis, muscle damage and elevated homocysteine after methionine loading, a recognised cardiovascular risk factor [2,4,5]. Despite its widespread occurrence in foods, dietary intake often falls below recommended levels, with requirements influenced by sex hormones and common genetic polymorphisms [1,3]. Contemporary nutritional and biomedical research continues to highlight choline’s relevance to neurodevelopment, cognitive performance, and healthy ageing, underscoring the need for reliable analytical and biosensing approaches in both clinical and research contexts [5,6].

While ACh has long dominated neurochemical analysis, free choline represents an analytically distinct and physiologically broader target. It exists across multiple matrices, including blood, cerebrospinal fluid, the extracellular space, and milk, with dynamic concentration ranges and variable turnover rates [2,7]. Accurate quantification is crucial for linking neurological activity to metabolic function, yet it remains technically challenging. Conventional methods such as high-performance liquid chromatography (HPLC; typically with pre- or post-column derivatisation for UV/fluorescence detection due to choline’s lack of a chromophore), capillary electrophoresis, and mass spectrometry (MS), including LC-MS/MS approaches that generally do not require derivatisation, instead using ion-pairing or hydrophilic interaction chromatography to improve retention and sensitivity, provide excellent selectivity [6,7] but require extensive sample handling and offer limited temporal resolution for in vivo monitoring [6,8]. These constraints have driven interest in electrochemical sensing technologies capable of real-time detection within micromolar physiological ranges [5,6].

**Figure 1:**
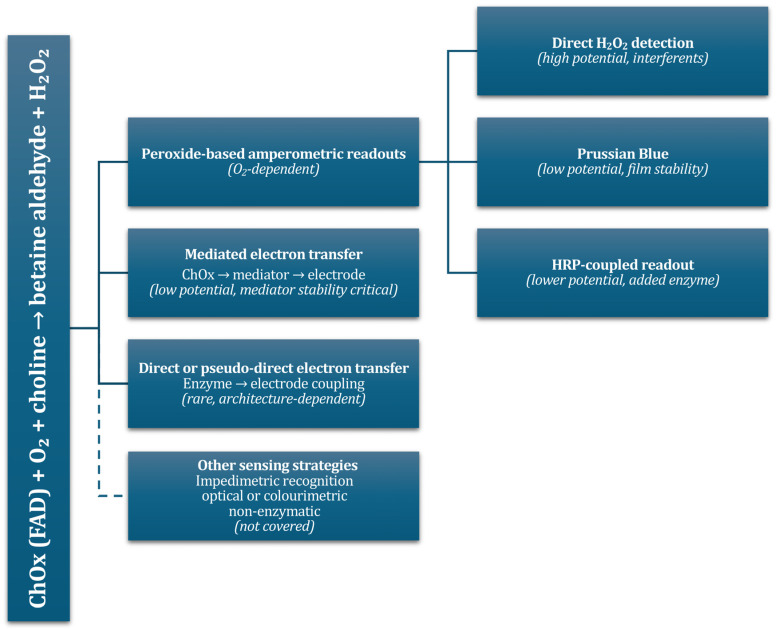
Overview of electrochemical choline-sensing strategies organised by signal generation mechanism. Choline oxidation by choline oxidase produces hydrogen peroxide, enabling peroxide-based amperometric readouts, while alternative pathways include mediated and direct or pseudo-direct electron transfer. Other sensing strategies are shown for context only.

Electrochemical sensors, especially those based on choline oxidase (ChOx), remain the workhorse for quantitative choline analysis owing to their simplicity, compatibility with miniaturisation and tuneable selectivity [8,9]. ChOx catalyses the oxidation of choline to betaine aldehyde with concomitant formation of hydrogen peroxide (H_2_O_2_), which underpins peroxide-based amperometric readouts and forms the basis of first-generation choline sensors. Subsequent developments have exploited alternative signal generation pathways, including mediated electron transfer (MET) using ferrocene, quinones or osmium-based mediators to enable operation at lower potentials, as well as direct or pseudo-direct electron transfer (DET) architectures that aim to establish electronic coupling between the enzyme and the electrode surface [5,6]. These distinct signal generation pathways, together with their associated design constraints, are summarised schematically in Figure 1. Choline detection has also been reported using molecular recognition and impedimetric or optical transduction strategies; however, as these rely on fundamentally different sensing principles, they are not discussed in detail in this review.

In parallel, advances in electrode engineering, including nanostructured transducers (e.g., graphene, carbon nanotubes (CNTs), nanoporous gold), permselective films and anti-fouling coatings, have further improved electron-transfer rates, stability and biocompatibility [6,7,9]. These developments have expanded the architectural space for choline sensing and enabled performance optimisation across a broader range of biological matrices.

Despite significant advances, the choline-sensing literature remains fragmented, with many reports emphasising materials or figures of metrics without clarifying how electron-transfer pathways shape selectivity and stability [6,8]. This fragmentation leaves unanswered questions about mediator choice, the credibility of DET claims for FAD-dependent enzymes, and the translation of benchtop prototypes to complex biological matrices [5,6,7]. Additionally, reporting standards remain inconsistent, which complicates performance comparisons across studies.

This review brings together literature published mainly between 2010 and 2025, drawing selectively on earlier biochemical and mechanistic work that lays the foundations for understanding enzyme function and electron transfer. Rather than providing an exhaustive catalogue of reported choline sensors, it offers a critical, mechanism-focused analysis of the architectures used to generate electrochemical signals. The emphasis is on the electron transfer pathways available to ChOx, oxygen-driven turnover, peroxide detection, and mediator-dependent mechanisms, alongside a careful evaluation of claims regarding DET and alternative non-enzymatic formats. By mapping each mechanism to its thermodynamic, kinetic and practical implications and aligning these with validation and reporting standards, the review establishes a coherent framework linking molecular-level design to reliable choline measurement across relevant matrices. Given choline’s importance in neurological, hepatic and nutritional biology, we also integrate target concentration ranges, interferent profiles and matrix-specific constraints to support translational development.

## 2. Choline Biology, Distribution and Analytical Requirements

### 2.1. Biological Roles, Matrices, and Typical Concentration Ranges

Choline participates in several essential metabolic pathways across mammals, but its physiological handling differs markedly between species and between tissues, creating distinct concentration regimes that are highly relevant for sensing (Table 1). In humans, choline homeostasis relies on hepatic uptake, release into the circulation bound to lipoprotein particles, and regulated transport into muscle and neural tissue via specific high- and low-affinity transporters [7]. These processes generate multiple biological compartments with different turnover kinetics, leading to significant variations in choline concentrations across plasma, cerebrospinal fluid (CSF), milk and the brain extracellular space (ECS) [1,2,3]. Appreciating both inter-species and compartmental differences is essential for defining realistic performance targets for electrochemical choline sensors.

In human biofluids, fasting adult plasma typically contains about 8 to 20 µM free choline (Table 1) [4,7]. Substantial intra-individual variability has also been reported, with fasting state, liver function, metabolic stress and recent dietary intake all contributing to shifts in plasma choline on the scale of several micromolar [4]. During pregnancy and early infancy, concentrations are substantially higher. For example, maternal and foetal or neonatal plasma often show six- to sevenfold increases relative to non-pregnant adults due to elevated phosphatidylcholine turnover and enhanced placental transport [1,2,3]. Placental tissue also contains large pools of choline-containing compounds because of its high phospholipid content [7,8]. In the literature, these measurements usually reflect total choline species rather than the free choline fraction. In human milk, choline is distributed between lipid-soluble forms, such as phosphatidylcholine and sphingomyelin, and water-soluble forms, including free choline, phosphocholine and glycerophosphocholine. The water-soluble fraction accounts for about 80–90% of total choline, with total concentrations rising from about 70 mg L^−1^ in colostrum to about 145 mg L^−1^ in mature milk, corresponding to approximately 700 to 1400 µM when expressed as free-choline equivalents [11,12].

Comparative animal studies highlight similar patterns with species-specific ranges. In rats, free choline averages about 11 µM in plasma and about 7 µM in cerebrospinal fluid [16]. In cattle, plasma-free choline typically ranges from 8 to 16 µM. In comparison, milk total choline (sum of free choline and glycerophosphocholine along with phosphatidylcholine-derived choline) ranges from about 500 to 900 µM depending on diet and lactation stage [20,21,22]. Canine studies show similar magnitudes, with dietary phosphatidylcholine supplementation increasing plasma choline concentration by approximately 4–5 µM over several weeks [24].

These data demonstrate that choline spans more than two orders of magnitude across biological matrices, from low micromolar levels in mammalian plasma to millimolar equivalents in milk. This breadth demands sensors with a wide dynamic range, matrix-specific calibration and adequate selectivity for co-present metabolites such as betaine and ethanolamine.

### 2.2. Temporal Dynamics, Turnover and Sampling Considerations

Choline turnover occurs far more slowly in systemic compartments than acetylcholine does at synapses. Outside the nervous system, choline flux is mainly governed by lipid metabolism and methyl-group donation, rather than by rapid neurotransmission. For example, changes in maternal choline intake alter milk choline concentrations over days to weeks rather than minutes [12]. In contrast, extracellular choline in the brain may fluctuate on much shorter timescales, as choline is a breakdown product of ACh hydrolysis and is influenced by cholinergic activity and transporter function [17,25].

From a sensor design perspective, this means measurement targets can range from steady-state monitoring (e.g., nutritional status, hepatic methylation) to transient fluctuations (e.g., extracellular choline in neuroscience). Accordingly, essential specifications include response time, drift stability, and dynamic range.

### 2.3. Interferent Profiles and Matrix-Matched Challenges

Across the targeted biological matrices, choline detection is complicated by co-present analytes and electroactive species. In plasma, typical interferents include ascorbate, urate and ethanolamine, with paracetamol contributing only following administration. In the brain extracellular space and CSF, ascorbate dominates the interferent profile, dopamine appears only in synaptic microdomains, and paracetamol likewise enters the compartment only after administration [6]. In milk and tissue homogenates, protein fouling, lecithin and phospholipid background, high salt and lipoprotein content create additional challenges [5]. Significantly, differences in ionic strength and fat content affect Prussian Blue (PB) and MET systems in distinct ways: PB is particularly sensitive to high ionic strength and surfactant-rich matrices, whereas MET performance is more influenced by lipid content and hydrophobic fouling. Viscosity shifts associated with ionic strength or matrix composition also modify the mediator’s diffusion coefficient, so the apparent sensitivity and response time change even when the enzyme layer remains unchanged. For example, in enzyme-based microelectrode studies, physiological ascorbate concentrations produced significant current offsets unless permselective membranes such as m-phenylenediamine (m-PD) or Nafion, or enzyme-free sentinel channels, were used [17]. These factors mean that sensor calibration, drift correction, selectivity panels and matrix-matched validation are essential rather than optional.

### 2.4. Implications for Sensor Specification

Considering the foregoing biological and analytical context, a choline sensor intended for translational applications must combine high sensitivity with stability across diverse biological matrices. The analytical limits should be tailored to the intended compartment: for neurochemical monitoring, the lowest physiological concentrations occur in cerebrospinal and brain extracellular fluid (approximately 1–3 µM), requiring a limit of quantification (LoQ) ≤ 1 µM and a limit of detection (LoD) proportionally lower to allow confident discrimination near this threshold [7,16]. For plasma, milk and foetal matrices, where concentrations span tens of micromolar to millimolar equivalents, the primary requirement is a broad, linear dynamic range extending to several hundred micromolar [11,12]. The required response time depends on the intended use: seconds for neurochemical monitoring of transient extracellular changes and typically seconds to a few minutes for nutritional or metabolic assessments [17,25]. This performance stands in contrast to conventional laboratory methods such as HPLC or LC-MS, which generally require much longer sample-to-result times. Selectivity must be sufficient to discriminate against common electroactive interferents, which are typically present at tenfold or greater excess, through permselective coatings or mediator engineering [6,8]. In addition, robust performance demands resistance to drift and biofouling, ensuring calibration stability over extended operation and preventing enzyme leaching during in vivo or ex vivo use [5]. Finally, practical deployment, particularly in point-of-care, requires minimal sample preparation and compatibility with real-time or near-real-time measurement formats [5,7].

## 3. Electron Transfer Pathways and Design Strategies for Enzymatic Choline Sensors

Electrochemical choline sensors converge on three fundamental modes of signal transduction: direct oxidation of enzymatically generated hydrogen peroxide, MET via artificial redox couples, and DET between the reduced flavin and the electrode surface. Understanding how film morphology, oxygen availability, mediator redox potential, electrode architecture, pH and temperature shape these pathways is essential for rational design. The following sections synthesise these mechanistic elements to provide a coherent framework for analysing and comparing enzymatic choline sensors across different transduction strategies.

### 3.1. Mechanistic Fundamentals

**Scheme 1:**
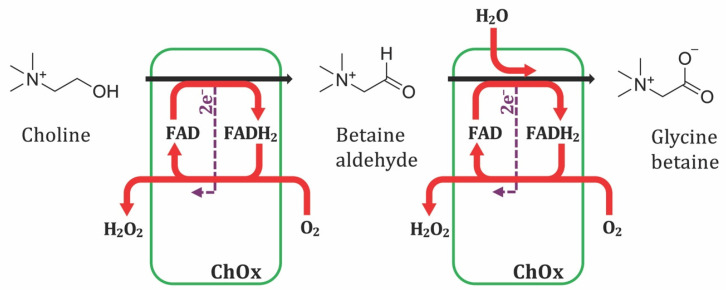
Two-step, four-electron oxidation of choline catalysed by choline oxidase (ChOx), producing two equivalents of H_2_O_2_ during flavin reoxidation by O_2_.

Choline oxidase contains the flavin adenine dinucleotide cofactor, FAD, and performs two sequential oxidations on choline (Scheme 1): first to betaine aldehyde and then to glycine betaine. In each step, the flavin is reduced to FADH_2_ and subsequently reoxidised by dissolved oxygen, producing H_2_O_2_. Under complete turnover, one molecule of choline yields two equivalents of H_2_O_2_ via two successive two-electron FAD reduction and oxygen-dependent reoxidation cycles [26,27,28,29,30].

Analytically, the theoretical electron count for complete choline turnover is four electrons per molecule. This corresponds to two peroxide equivalents under oxygen turnover or, in mediated systems, either two mediator turnovers for a two-electron mediator or four turnovers for a one-electron mediator. Many reports nonetheless treat the process as effectively two electrons, either by assuming formation of one equivalent of H_2_O_2_ per choline or because only the first flavin cycle is expressed within the measurement timescale. In thick films or under low-oxygen conditions, reoxidation of FAD by oxygen can become rate-limiting, so the recorded signal reflects a mixture of two- and four-electron behaviour determined by transport and kinetics. This is benign for empirically calibrated devices, where the slope incorporates the operative stoichiometry, but becomes critical for calibration-free, ratiometric or coulometric formats and for any method that embeds a fixed electron count in Faraday’s law [31,32,33,34,35,36]. If the sensor assumes two electrons per choline while the operative stoichiometry drifts with oxygen tension, film thickness, or measurement time, the inferred concentration becomes biased and matrix-dependent.

For this reason, meaningful comparison of sensitivities and slopes across architectures requires explicit control or reporting of film thickness, oxygen availability and measurement timescale. Designs intended to assign or fix the electron number should either enforce conditions that express both flavin cycles, such as thin hydrated films with adequate oxygen or a validated mediated pathway that outcompetes oxygen, or treat the electron count as an experimentally verified parameter confirmed through air-saturated and deaerated calibrations, catalase- and mediator-absent controls, and time-based tests that confirm the expected charge-to-mass relation.

In immobilised architectures, choline, oxygen, and, when present, artificial mediators must diffuse through hydrated polymer-enzyme matrices before reaction occurs. Numerous studies have shown that the measured current reflects a mixed kinetic regime governed by both enzymatic turnover and transport limitations [10,37,38,39]. A convenient description adapted from classical oxidase film models expresses the product flux, *J*, as a harmonic sum of a kinetic term and a diffusive term [40,41]:

$$I=nFAJ=nFA{\left[\frac{1}{\frac{1}{k_{cat}{\left[ChOx\right]}_{imm}}+\frac{\delta^{2}}{2D_{app}[Ch]}}\right]}^{-1}$$

where *δ* is the effective film thickness, *D*_app_ the apparent diffusion coefficient for choline in the matrix, *k_cat_* the catalytic turnover number of immobilised ChOx, [ChOx]_imm_ the effective concentration of active enzyme in the film, [Ch] the local choline concentration within the matrix, *n* the electrons transferred per choline molecule under the operative stoichiometry (e.g., 2 or 4 electrons depending on whether one or both flavin cycles contribute to the detected H_2_O_2_), *F* the Faraday constant, and *A* the electroactive electrode area. Thin, highly hydrated films drive *J* toward kinetic control, whereas thicker or densely cross-linked layers push the response toward diffusion-dominated behaviour.

**Scheme 2:**
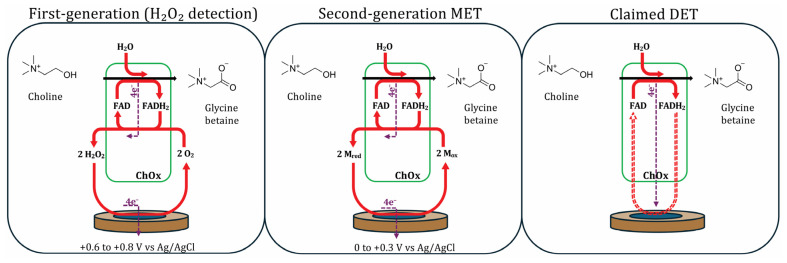
Comparison of the three electron transfer pathways in ChOx-based choline sensors: first-generation H_2_O_2_ detection, second-generation mediated electron transfer (MET), and claimed direct electron transfer (DET). In MET, the oxidised mediator (M_ox_) accepts electrons (cartoon representing a two-electron oxidation/reduction mediator) directly from FADH_2_, becoming reduced (M_red_) and thereby replacing O_2_ as the oxidant at the enzyme level.

Electron flow from the enzyme to the electrode proceeds through three mechanistic pathways that underpin all ChOx-based choline sensors (Scheme 2) [8,42,43]. In first-generation systems, the signal arises from oxidation of enzymatically generated H_2_O_2_ during flavin reoxidation, which typically requires high anodic potentials (+0.6 to +0.8 V vs. Ag/AgCl, 3 M NaCl) and exposes the measurement to co-oxidation of common interferents such as ascorbate, urate, dopamine and paracetamol [9,26,37]. Second-generation MET architectures couple the reduced flavin directly to artificial mediators, most commonly ferrocene derivatives, quinones, phenothiazines, or osmium redox-polymer networks, which relay charge to the electrode at substantially lower bias (ca. 0.0 to +0.3 V vs. Ag/AgCl, 3 M NaCl), thereby improving selectivity and, when properly implemented, reducing oxygen dependence at the enzyme level [42,43]. Importantly, MET is mechanistically distinct from PB and other H_2_O_2_-electrocatalytic strategies: MET mediators oxidise FADH_2_ and replace O_2_ in the enzymatic cycle, whereas PB operates only at the electrode, electrocatalytically reducing H_2_O_2_ that has already formed and therefore does not change the enzyme’s oxygen requirement or suppress H_2_O_2_ generation. The third pathway, often labelled DET, posits the oxidation of ChOx(FADH_2_) at the electrode, but structural and kinetic evidence strongly contradicts this interpretation (see below) [28,29,30,44]. The FAD isoalloxazine ring is deeply buried and protein-shielded, placing it beyond practical electronic coupling distances, and kinetic studies consistently show obligatory, O_2_-dependent reoxidation rather than any protein-electrode pathway [28,29,30,44]. These mechanistic distinctions are crucial for interpreting sensor behaviour and for understanding how applied potential, selectivity and oxygen availability shape analytical performance across architectures.

Because ChOx(FADH_2_) can be reoxidised by either O_2_ or an artificial mediator, oxygen competition must be considered not only in first-generation sensors but also in MET architectures that have not demonstrated oxygen-independent turnover, namely systems in which mediator transport or regeneration is insufficient to outcompete oxygen. In thin, well-hydrated films, O_2_ typically re-oxidises FADH_2_ fully within the measurement window, so both reductive half-reactions are expressed, and the full peroxide yield is obtained. In thicker or highly cross-linked matrices, however, the effective O_2_ diffusivity can drop by one to two orders of magnitude relative to bulk, and only a fraction of FADH_2_ is reoxidised in time. Under these conditions, the apparent peroxide yield per choline decreases toward one equivalent, and mediator turnover begins to compete directly with oxygen for access to the reduced flavin.

Direct O_2_ profiling (microelectrodes), stirred-quiescent comparisons, and controlled-atmosphere calibrations consistently show that choline-sensing films operate near the O_2_-limitation threshold, especially when immobilisation layers exceed ca. 1–2 µm or when mediators compete with O_2_ for FADH_2_ [10,39,41]. Nanostructured supports (e.g., CNTs, graphene, porous Au) can raise internal porosity and improve access, partially alleviating O_2_ constraints, but they do not eliminate oxygen dependence.

In MET systems, the fraction of turnover carried by mediator rather than oxygen is set by the mediator’s redox potential, concentration, mobility within the film and the efficiency with which it is regenerated at the electrode [39,41]. When the flux of oxidised mediator to the enzyme is high, MET can largely suppress oxygen limitation by replacing oxygen as the primary oxidant of FADH_2_; when it is low, oxygen continues to re-oxidise a substantial fraction of FADH_2_, and the response remains oxygen dependent. Accordingly, oxygen availability must be treated as an explicit design variable rather than a passive background condition. More broadly, oxygen acts not only as a co-substrate but also as a rate-limiting reagent: its transport sets the effective electron count, inflates or depresses apparent Michaelis-Menten parameters, and influences the stability of ChOx-based films across biological matrices.

Taken together, these mechanistic elements define the operational boundaries of ChOx-based choline sensors. The two-step FADH_2_ mechanism establishes the theoretical electron yield; film-confined kinetics determine whether both reductive half-reactions are expressed within the measurement window; mediator selection fixes the working potential, selectivity, and oxygen dependence; and the structural constraints of ChOx render DET implausible. These factors, acting together, shape sensitivity, linear range, drift behaviour, and calibration requirements across first-generation, MET, and claimed DET systems, providing the foundation for the detailed analysis of each electron transfer mode in the sections that follow.

### 3.2. First-Generation Sensors

First-generation choline sensors detect the H_2_O_2_ formed when ChOx(FADH_2_) is reoxidised by dissolved oxygen. This was the earliest and remains the most widely used configuration of ChOx-based electrodes, providing the foundation for later design innovations [9,42]. After enzymatic turnover, H_2_O_2_ diffuses from the ChOx layer to the transducer, where it is oxidised at high anodic potentials (Scheme 2) [10,37]. The resulting current is proportional to the H_2_O_2_ production rate and, under non-limiting oxygen, reflects the enzymatic oxidation of choline [26,27]. Because H_2_O_2_ oxidation is a two-electron process, these devices typically report an apparent two-electron stoichiometry, even though complete biochemical turnover of choline yields two H_2_O_2_ per substrate (i.e., four electrons per choline molecule) [26,28,44]. This disparity becomes analytically important whenever film thickness, oxygen availability, or the measurement time prevents full expression of both reductive half-reactions, in which case calibration slopes reflect only a fraction of the theoretical four-electron ceiling [10,39].

First-generation sensors offer large signal amplitudes and straightforward construction and have historically yielded substantial insight into choline dynamics in the extracellular space and in brain microdialysate (Table 2) [9,33]. Their key limitation is the high anodic potential required for H_2_O_2_ oxidation, which renders them vulnerable to the co-oxidation of endogenous electroactive molecules, such as ascorbate, urate, dopamine, and paracetamol [9,37]. Without permselective barriers, for example, Nafion, electropolymerised m-PD or overoxidised polypyrrole, these interferents generate large background currents and degrade quantitative accuracy. Even with barriers, long-term stability depends on careful control of film thickness, hydration, and fouling in complex matrices [9,37].

It is also important to note that many of the performance metrics in Table 2 were determined under buffered laboratory conditions. Although such calibrations are essential for characterising device behaviour, they often overestimate the device’s actual sensitivity in the real world. Complex biological matrices introduce adsorption, fouling, ionic strength effects, and competition from endogenous electroactive species, which alter the effective LoD, LoQ, and linear range. Several reports have extrapolated in-buffer performance to plasma, cerebrospinal fluid, microdialysate or milk without demonstrating that these matrix-dependent effects are controlled, leading to optimistic claims of biological applicability. Consequently, buffer-based optimisation should be interpreted as necessary but not sufficient evidence for reliable sensing in complex media.

As Table 2 shows, first-generation formats encompass both in vivo and in vitro implementations, but all derive their signal from the electro-oxidation of enzymatically generated H_2_O_2_. Selectivity and operating potential can be improved by introducing electrocatalysts for H_2_O_2_ at the transducer. Platinum or gold nanostructures, carbon nanomaterials, and PB each lower the overpotential for H_2_O_2_ transformation and thereby suppress co-oxidation of interferents (Table 2) [37,43,57]. Crucially, these modifiers act only at the electrode: they catalyse the electrochemical step without altering the ChOx catalytic cycle. Prussian blue, often dubbed a “synthetic peroxidase,” is especially useful because it enables H_2_O_2_ detection at 0.0 V vs. Ag/AgCl, 3 M NaCl, thereby markedly improving resistance to endogenous interferents [57]. However, because none of these catalysts interacts with FADH_2_, the enzyme’s oxygen dependence and the biochemical stoichiometry of H_2_O_2_ formation remain unchanged [26,27,44].

First-generation systems remain sensitive to fluctuations in oxygen because O_2_ availability directly controls the rate of H_2_O_2_ production [10,39]. In complex sample environments, endogenous interferents, fouling of permselective coatings, and gradual shifts in film hydration or cross-linking can each contribute to baseline and sensitivity drift over time [37,38]. Even so, their simplicity, large signal amplitudes, and mature microfabrication workflows keep H_2_O_2_-mode devices useful both as practical analytical tools and as a benchmark against which second-generation and purported DET formats are evaluated [9,43].

### 3.3. Second-Generation Sensors—Mediated Electron Transfer

Second-generation choline sensors use a redox mediator to oxidise ChOx(FADH_2_) and regenerate the mediator at the electrode under low bias, decoupling turnover from oxygen and lowering the working potential (Scheme 2). Two experimentally validated architectures illustrate the design space. In diffusional-mediator formats, benzoquinone oxidises FADH_2_ in solution and is reoxidised at approx. +0.45 V vs. Ag/AgCl, 3 M NaCl, providing oxygen-independent turnover over 0.125–1.25 mM choline. Because benzoquinone enters the system already in its oxidised form, applying +0.45 V does not further oxidise it. The enzyme instead reduces benzoquinone to hydroquinone, and the electrode detects this directly by oxidising hydroquinone back to benzoquinone. The signal therefore arises from the accumulated reduced mediator rather than from a catalytic regeneration cycle, which only occurs when the mediator is electrochemically recycled in situ, as in immobilised ferrocene systems. However, the potential remains within the interferent window and requires permselective barriers [58]. In immobilised-mediator approaches, ferrocene confined within conductive nanocarbon scaffolds supports low-bias catalytic turnover (operated here at −0.13 V vs. Ag/AgCl, 3 M NaCl) despite its positive formal potential, as rapid in-film regeneration minimises the required overpotential. The system delivers a linear range of 1–400 µM, LoD = 0.35 µM, and ca. 8 s response time [59].

A successful mediator must place its formal potential slightly more positive than the FAD/FADH_2_ couple to provide driving force yet remain within a low-bias window. Structure-activity studies on flavin-reactive quinones show that compact, neutral mediators with suitably tuned potentials react fastest with FADH_2_, whereas bulky or highly charged mediators are hindered by desolvation penalties and restricted access to the narrow, largely apolar flavin channel [60,61]. Short tunnelling distances are critical: distance-dependent studies consistently show exponential penalties for both enzyme-to-mediator and mediator-to-electrode transfer, favouring thin, hydrated films and immobilisation environments that maintain proximity and mobility [62,63,64].

Mediator performance must be demonstrated experimentally rather than inferred. Oxygen independence requires air/deaerated calibrations, mediator-absent controls, heat-denatured enzyme tests, and catalase additions for positive-potential operation [27,65]. Because film thickness and oxygen access can shift the effective electron count between two and four electrons per choline, the assumed stoichiometry must be stated and, where possible, verified by charge-mass tests or catalase experiments [31,62].

Charged mediators, although fast at bare electrodes (e.g., ferricyanide, [Ru(bpy)_3_]^2+/3+^), rarely function as true inner-sphere relays in immobilised ChOx films. Their strong solvation, film-charge interactions, and poor access to the hydrophobic flavin channel mean they typically report film permeability rather than authentic flavin-mediated effects [62,66,67,68,69,70]. By contrast, compact neutral relays such as benzoquinone and immobilised ferrocene repeatedly deliver efficient, low-noise MET in ChOx-only architectures [58,59].

Ultimately, mediator identity, redox potential and immobilisation strategy must be optimised together, as none can compensate for deficiencies in the others. When these elements are coherently matched, mediated electron transfer enables genuinely low-bias, oxygen-independent operation. It remains the most robust and practical alternative to peroxide detection or the structurally unrealistic claims of direct electron transfer.

### 3.4. Direct Electron Transfer with Choline Oxidase

DET is attractive because it promises oxygen-independent turnover at very low applied potential, but the structural characteristics of ChOx render genuine DET intrinsically unlikely. The flavin cofactor sits deeply buried within a narrow, largely apolar access channel, placing the redox centre several nanometres from any electrode surface. Classical interfacial studies show that tunnelling rates fall exponentially with distance, making productive coupling impractical without engineered relays positioned within tunnelling range of the flavin [62,63,64,71]. Consistent with this, kinetic and structural analyses of ChOx demonstrate obligatory O_2_-dependent reoxidation rather than any detectable protein-electrode pathway [28,29,30,44].

Most reports of DET-like behaviour resolve to mediated or peroxide-driven artefacts. Apparent low-bias currents on CNTs, graphene or nanoporous carbons often collapse under nitrogen, disappear upon catalase addition, or persist only when residual mediators are present, indicating H_2_O_2_ electrocatalysis or leached redox species rather than direct flavin oxidation [62,65]. High-surface-area materials can amplify such artefacts through area effects, film-dependent partitioning, or adsorption of trace mediators.

A credible DET claim, therefore, requires stringent controls: (*i*) identical currents under air and nitrogen; (*ii*) insensitivity to catalase; (*iii*) complete loss of signal upon mediator removal; (*iv*) enzyme-free and heat-denatured controls; and (*v*) potential windows that exclude H_2_O_2_ electrocatalysis and common interferents. Transport signatures should correspond to interfacial enzymatic kinetics rather than to peroxide diffusion or mediator movement, as verified by area scaling, film thickness series, and rotating-electrode tests [62,64].

Although theoretical pathways to DET exist, such as positioning conductive centres within tunnelling distance via nanoporous gold or oriented coupling along the substrate access path, no study to date has provided conclusive evidence satisfying all DET criteria for ChOx [62]. Contemporary enzymology and bioelectrocatalysis reviews therefore regard ChOx as an enzyme that requires either oxygen or an artificial mediator for turnover, with reliable low-potential operation achieved only through MET or bienzymatic HRP wiring [9,62,63,64,65,72].

In practice, DET should not be invoked without full supporting controls, as many reported signals collapse under proper mechanistic testing.

### 3.5. Bienzymatic Choline Oxidase and Horseradish Peroxidase

**Scheme 3:**
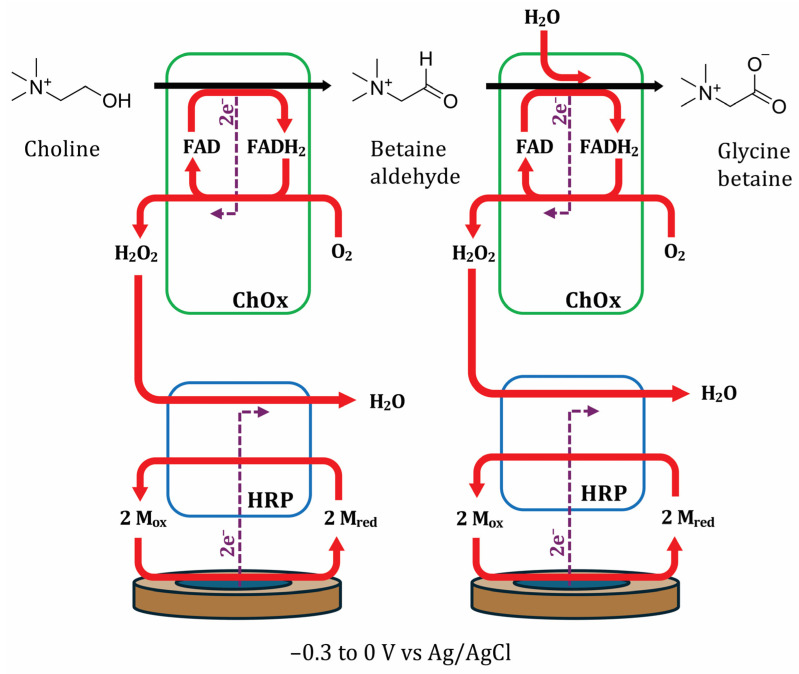
Bienzymatic ChOx-HRP operation for a one-electron mediator. Each H_2_O_2_ molecule generated by ChOx oxidises two mediator equivalents via the HRP catalytic cycle.

Bienzymatic choline sensors (Scheme 3) couple ChOx with horseradish peroxidase (HRP) so that the H_2_O_2_ generated by the oxidase is consumed at or near the sensing interface, while the redox state of the peroxidase is interrogated at low applied potential through a mediator that cycles at the electrode. This architecture can be implemented with both enzymes co-immobilised on the transducer, with one or both enzymes soluble in a stirred or flow compartment adjacent to the electrode, or within microfluidic channels that deliver the oxidase product to an HRP-mediator layer [18,73,74,75,76,77,78]. Under complete turnover, ChOx performs two FAD reductions and generates two equivalents of H_2_O_2_ per choline, which HRP reduces by forming its high-valent intermediates that oxidise the mediator [77]; the electrode then reduces the oxidised mediator at a potential near zero or at modest cathodic bias (negative potentials). The measured charge, therefore, corresponds to two electrons per peroxide and four electrons per choline.

Effective operation requires that HRP is not rate-limiting for the peroxide flux from ChOx. Studies of oxidase-peroxidase cascades consistently show that the peroxidase must be present in kinetic excess so that the oxidase step remains rate-determining [79,80]; otherwise, peroxide accumulates, delays the response, and reduces the attainable activity. In the glucose oxidase (GOx)-HRP bienzymatic system, optimal performance was obtained only when the peroxidase activity exceeded the oxidase by a factor of about three, corresponding to a molar excess of approximately five to ten, and larger excesses were required when mass transport limitations were present or when the enzymes were immobilised in separate domains [79,80]. These results generalise to oxidase-peroxidase formats more broadly and align with standard practice in flavoprotein oxidase assays, where the peroxidase is routinely supplied in excess to avoid a bottleneck at the reporting step [79,80].

The mediator that couples the electrode to HRP is a key design element, and Table 3 summarises representative low-bias systems. Osmium-based redox polymers, ferrocenyl nanocomposites, phenothiazine derivatives, poly(3,4-dihydroxythiophene) (PTH), and related conducting films have all been used to regenerate HRP at low potential, with the mediator chemistry determining the accessible potential window, turnover rate, resistance to leaching and compatibility with complex matrices [59,73,74,76,78,81,82,83]. Beyond the ChOx-HRP formats listed here, other mediators, such as ferrocyanide, hydroquinone derivatives and additional ruthenium or osmium complexes, have been used to wire HRP in sensors based on different oxidases, illustrating the broader design space available for bienzymatic architectures [59,75,77,78,81]. Flow-assisted formats and wired peroxidase chemistries provide fast and stable readouts and remain the most reliable means of achieving low-potential choline detection. In practice, bienzymatic choline sensors are most robust when the oxidase loading and transport of choline and oxygen define the analytical range. At the same time, HRP and mediator chemistry are tuned to ensure rapid peroxide turnover without accumulation or inactivation.

### 3.6. Operational pH Window for ChOx with and Without HRP

**Figure 2:**
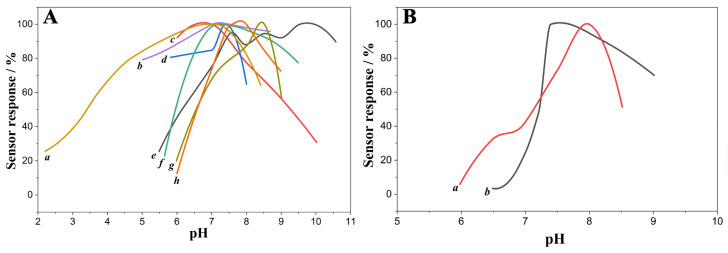
Dependence of choline sensor response on buffer pH, with each trace normalised to its own maximum (100%). (**A**) Single-enzyme ChOx-modified electrodes, using (*a*) ChOx/TGA-SAM/chip [84], (*b*) ChOx/IL/NH_2_-MWCNTs/GC [85], (*c*) ChO-TBO-PPy-PVS/Pt [86], (*d*) ChOx/Ga_2_O_3_ NWs/CB/CSPE [87], (*e*) ChOx-poly(m-PD)/Pt [88], (*f*) ChOx-SBA-15/TD-p-AgSA [89], (*g*) ChOx/GNP/MWCNT/GC [54], and (*h*) ChOx-IL-PB-BL-MWCNTs/GC [46] as the sensor; (**B**) bienzymatic ChOx-HRP-modified electrodes, using (*a*) ChOx-HRP-PMG-CNF-MnO_2_/SPCE [73] and (*b*) ChOx-HRP/CPMEs [76] as the sensor.

Enzyme kinetics and device transport jointly set the observed pH dependence of ChOx-modified electrodes. Figure 2 presents normalised current-pH responses for single-enzyme ChOx sensors (Figure 2A) and bienzymatic ChOx-HRP stacks (Figure 2B). Across the single-enzyme traces, the response rises from a mildly acidic buffer, reaches a broad maximum between about pH 7.0 and 8.2, and then declines at higher pH. The spread of the peak by as much as one pH unit across studies is expected because film hydration, ionomer loading, mediator identity and oxygen access vary, and these factors control whether both FAD reduction cycles are expressed within the measurement time. Devices that use thin hydrated layers and mediated readout at low bias tend to peak closer to physiological pH, whereas thicker or highly cross-linked films shift the apex upward and narrow the window.

Taken together, the device data define an operational pH window centred near neutral to mildly alkaline conditions, in agreement with pH-rate profiles measured for the isolated enzyme [27,90], which showed that the catalytic base must be deprotonated for turnover. This gives an apparent pKa near eight for the step that limits *k_cat_* and *k_ca_*/*K*_M_ under steady-state conditions, where *K*_M_ is the Michaelis constant corresponding to the substrate concentration that gives half-maximal velocity [27,90]. This places the intrinsic kinetic optimum for ChOx around neutral to slightly alkaline pH, consistent with the maxima in Figure 2A. The fall above about pH 8.5 seen in several curves is therefore most plausibly extrinsic to the chemical step at the flavin, arising from reduced oxygen solubility and diffusion in the film, altered ionisation of surface residues that govern access to the buried cofactor, and mediator stability under alkaline conditions rather than a shutdown of the hydride transfer step [90]. The assignment of an essential catalytic base is further supported by mutational work implicating His466 and neighbouring residues in proton management at the active site, providing a structural rationale for a neutral-lean optimum [28,30].

When HRP is added to consume peroxide and to shuttle charge at low or negative bias, the composite response usually retains a neutral-centred maximum. Still, the usable window can broaden slightly, and the shoulder toward pH 7 becomes more pronounced. In Figure 2B, where HRP is wired through fast mediators, the maxima lie near pH 7.6 to 8.0, and the decline toward alkaline pH is delayed relative to some single-enzyme traces. This behaviour is consistent with the enzyme-level stability map, which showed that ChOx activity is highest near pH 8 with progressive inactivation occurring only outside a broad window spanning roughly pH 6 to 9, and with faster loss in more extreme acidic or alkaline conditions [91]. Because the oxidase step remains dependent on dissolved oxygen, the bienzymatic stack retains the oxygen-limited behaviour of ChOx, even though the HRP layer shifts the electrochemical readout to low bias. The sharper losses above pH 8.5 in Figure 2B are therefore consistent with the extrinsic alkaline effects noted earlier, including reduced oxygen solubility, altered ionisation of access residues and mediator instability, and may also include a contribution from HRP, which is known to lose activity under alkaline conditions. In bienzymatic ChOx-HRP stacks, the operational pH optimum must therefore reflect the stability of both enzymes.

Two practical implications follow for method development and reporting. First, treat the pH optimum as a property of the finished stack rather than the purified enzyme. Report the full device response curve with buffer composition, ionic strength and temperature, and confirm that the four-electron yield per choline is expressed at the chosen pH when oxygen is not limiting. Second, in bienzymatic formats, confirm that the HRP mediator remains fast and chemically stable in the same buffer in which ChOx performs best; otherwise, a mediator bottleneck can mimic a shift in the oxidase optimum. Overall, Figure 2 aligns with the catalytic base model and the stability window [27,90,91], providing enzyme-level support for selecting a working pH near 8 for ChOx alone and slightly lower, around pH 7.5–8, for bienzymatic ChOx-HRP systems to preserve HRP activity.

### 3.7. Operational Temperature Window for ChOx with and Without HRP

**Figure 3:**
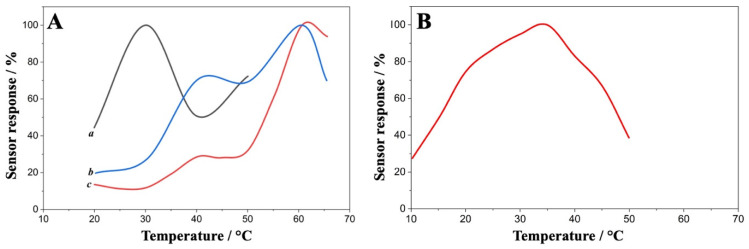
Temperature dependence of choline sensor response, with each trace normalised to its own maximum (100%). (**A**) Single-enzyme ChOx-modified electrodes, using (*a*) ChO-TBO-PPy-PVS/Pt [86], (*b*) ChO-PPy-PVS/Pt [92], and (*c*) ChOx-PANI-SiO_2_/CPE [93] as the sensor; (**B**) Bienzymatic ChOx-HRP-modified electrodes operated at low or negative bias using ChOx-HRP/CPMEs [76] as the sensor.

Temperature affects both the intrinsic turnover of ChOx and the transport terms that shape the current, including oxygen solubility, mediator kinetics and film hydration. When responses are normalised to each device’s maximum, the single-enzyme traces in Figure 3A exhibit configuration-dependent patterns. One architecture displays a narrow maximum around 30 °C, followed by a marked decline to about 40 °C [86], whereas two other architectures show broader optima centred near 40 °C and fall above roughly 45 °C [92,93]. Reports of apparent peaks close to 60 °C should be interpreted as responses of the materials stack rather than evidence that the enzyme operates best at that temperature; warming can reduce viscosity, enhance mediator mobility or polymer conductivity, and alter wetting or porosity in ways that temporarily increase current more rapidly than the enzyme loses activity. This does not indicate an elevated catalytic optimum for ChOx itself.

Solution-based measurements show that ChOx turnover increases with temperature through the low thirties and then declines as thermal inactivation becomes significant, with activity and half-life both falling above roughly 40 °C under typical assay conditions [27,28,30,90,91]. These intrinsic kinetics align with the device-level behaviour in Figure 3A and support the interpretation that responses above ~45 °C reflect temperature-dependent changes in the material stack rather than an elevated catalytic optimum.

For bienzymatic architectures that couple ChOx to HRP (Figure 3B), the composite temperature profile typically centres in the 30–35 °C range and narrows relative to that of single-enzyme devices. An apparent decline is generally observed above ca. 40 °C. This behaviour reflects the peroxidase step becoming limiting as temperature increases and the relatively rapid loss of HRP activity and structural stability in this region [94,95,96].

From a practical standpoint, sensors intended for physiological use should be calibrated and operated under controlled temperature conditions that match the intended application, typically near 37 °C for human or mammalian biofluids. Because device-level performance often begins to drift above about 35 °C due to changes in film hydration, mediator mobility and oxygen transport, calibrations at 37 °C should be carried out using thin, well-hydrated coatings and fully matched buffer conditions. When unusually high apparent optima are reported, enzyme integrity after heat exposure should be verified, the temperature response of the reference electrode should be checked, and catalytic and transport contributions should be disentangled by repeating calibrations with viscosity-matched buffers. Overall, across device formats, the reliable functional window of ChOx-based choline sensors lies between about 30 and 35 °C, and operation at 37 °C is feasible when the film architecture is designed to avoid thermal artefacts.

### 3.8. Non-Enzymatic and Neutral-pH Strategies

Enzyme-free choline sensing at neutral pH is best established in potentiometry. Ion-selective electrodes (ISEs) that embed molecular receptors for the choline cation in plasticised membranes, most commonly cavitands, calixarenes, or porphyrinoids, offer near-Nernstian slopes with micromolar or sub-micromolar detection limits, low-bias operation, and good medium-term stability. A representative solid-contact sensor utilising an octaamide cavitand on a carbon nanotube transducer exhibited a slope of 57.3 ± 1.0 mV per decade, a dynamic range spanning 10^−5^ to 10^−1^ M, and an LoD of 0.40 µM, with good medium-term stability [97]. A more recent calixarene-based membrane, paired with a sulfonated calixarene in the inner solution to suppress transmembrane flux, reported a linear range from 0.03 µM up to 1 mM, an LoD of 0.061 µM, and response times typically under 5 to 60 s in food matrices, while remaining enzyme-free and compatible with neutral media [98]. Porphyrinoid ionophores have also been explored; an all-solid-state protoporphyrin-based choline ISE reported a 1 µM to 1 mM linear range with an approximate LoD of 0.5 µM in artificial cerebrospinal fluid [99]. These devices are inherently oxygen-independent, eliminating the need to handle peroxide. Still, they must manage discriminating power against structurally similar quaternary ammonium interferents (e.g., acetylcholine, carnitine, and betaine), ionic strength effects on phase-boundary potentials, and rigorous potential referencing to limit drift during long recordings [97,98,99].

Beyond potentiometry, genuinely non-enzymatic redox detection of choline at physiological pH is rare. One notable approach utilises a nitroxyl radical organocatalyst, nortropine N-oxyl (NNO), as a homogeneous redox mediator that electrocatalytically oxidises choline at pH 7.4, enabling amperometric measurement without ChOx in phosphate buffer over the range 0.1–10 mM [100]. While promising as proof-of-concept, this chemistry has not yet been validated in complex matrices for selective choline quantification and, to date, remains far less mature than ionophore-based potentiometry [100].

In summary, at neutral pH, the only mature non-enzymatic route for choline is potentiometric recognition with ionophore membranes; amperometric organocatalysis remains at the proof-of-concept stage. Until a selective, oxygen-independent redox pathway is validated in complex matrices, these ion-selective platforms set the practical benchmark for enzyme-free choline analysis. Future gains are likely to come from host architectures that provide greater selectivity against quaternary ammonium interferents, more stable solid-contact transduction and referencing to minimise drift, and rigorous matrix-matched validation so that figures of merit are directly comparable to those of enzymatic stacks.

## 4. Platforms and Form Factors (Matrix-Matched)

Choline sensing performance depends as much on sampling format and device geometry as on the electron transfer pathway. In brain and other biological matrices, transport, biofouling, and interferent co-profiles vary widely, so sensor stacks must be tuned to the sampling method and to the form factor that will carry them in use.

Microdialysis coupled with amperometry provides a robust approach for matrix-matched calibration and extended in vivo and in vitro recordings [18,33]. Choline oxidase, peroxidase, or mediated electron-transfer stacks can be placed downstream of the probe, allowing ready replacement of the detector while the sampling interface remains constant. Probe recovery, which determines effective sensitivity, is governed primarily by perfusion rate and membrane geometry. At 1 to 2 µL min^−1^ with 2 to 4 mm membranes, relative recovery for small hydrophiles such as choline typically ranges from 10 to 40%, and it decreases further as perfusion rates increase or membrane walls thicken, which lengthens transients but improves stability [18,33]. Reporting concentrations normalised to in situ recovery avoids false comparisons across studies. These systems favour low-bias detection with permselective barriers to suppress ascorbate and urate and benefit from ratiometric or self-referenced normalisation to correct for probe-to-probe variability [17,32].

Self-referencing microelectrode arrays place sentinel channels adjacent to choline channels to subtract interferents and drift in real time. Ceramic or carbon microarrays coated with ChOx and a permselective overlayer have been used in rat cortex to track tonic choline at second-scale resolution while suppressing crosstalk and motion artefacts [32]. Accurate subtraction depends on matching the barrier stack and geometric area between the sentinel and the choline channel; even small mismatches in the thickness of the poly(m-PD) barrier or in Nafion loading produce baseline curvature that leaks into the differential trace [17,32,37]. This geometry is well-suited for the first-generation peroxide mode and for oxygen-independent mediated electron transfer at low potentials, provided that the reference channel is closely matched in geometry and barrier chemistry [17,32].

Carbon fibre microelectrodes with enzyme coatings trade ease of fabrication for a very small geometric area and fast mass transport [19]. When combined with thin poly(m-PD) or Nafion topcoats, they deliver short diffusion paths, low background and good resistance to fouling [19,25,57]. Because the oxygen-dependent reoxidation of FADH_2_ can limit turnover in thicker coatings, maintaining thin, well-hydrated films allows full four-electron expression per choline while permitting operation at a lower potential when a mediator or PB is used. These devices have been used extensively for choline and for ACh via acetylcholinesterase and ChOx cascades, with controls to avoid peroxide artefacts and to confirm oxygen sensitivity where relevant [19,25,57].

Chip-level transducers broaden the stack options. Organic electrochemical transistors read the redox product at the gate and operate at very low bias; however, for cholinergic analytes, they still rely on enzyme chemistry and peroxide generation at the interface, so they belong with enzymatic architectures rather than non-enzymatic routes [101]. Screen-printed platforms support large-area permselective and catalytic films such as PB for near-zero-potential peroxide readout or immobilised ferrocene and quinone relays for mediated electron transfer, and they enable disposable formats for food and serum analysis [39,43,46,50,53]. For disposable chips, select the readout window before selecting the chemistry: PB for near-zero-potential peroxide reduction in complex serum or food matrices; immobilised ferrocene or quinone for oxygen-independent mediated transfer at 0.0 to +0.3 V with thin, hydrated enzyme films; and bienzymatic ChOx-HRP for operation at 0.0 to −0.3 V, where a fast HRP mediator shuttles charge at negative bias while peroxide is consumed within the stack.

Taken together, the sampling interface and device geometry are as decisive as the electron transfer pathway. Thin, hydrated films on small-area transducers or low-residence-time microdialysis favour full expression of both FAD cycles, while arrays reduce drift when the sentinel and choline channels are truly matched in barrier and area. Disposable chips work best when the potential window is set first, then the chemistry is chosen to fit it. For a fair comparison across studies, report the reference electrode used and, where necessary, convert potentials to a standard scale; also report barrier composition and thickness, microdialysis recovery (where applicable), and the core controls outlined earlier. With these elements in place, the choice among formats can be driven by matrix concentration, allowable bias, selectivity needs, and the practicality of routine calibration.

## 5. Validation and Reporting Standards

Electrochemical choline sensors can be meaningfully compared only when the operative electron transfer pathway is correctly identified. Although the preceding sections outline peroxide-based, mediated and bienzymatic formats, the practical difficulty lies in recognising which mechanism is active in a given device and how film structure, oxygen transport and mediator behaviour shape the recorded current. This section consolidates the mechanistic signatures into a concise diagnostic framework.

Peroxide-based readout is indicated by three features: signals that appear only at positive potentials where H_2_O_2_ is oxidised; a strong dependence on dissolved oxygen; and a stoichiometry of one to two peroxide equivalents per choline, shaped by film thickness and oxygen diffusion. Permselective barriers reduce but do not eliminate co-oxidation of endogenous interferents. Appropriate controls include air vs. nitrogen measurements, catalase addition and thickness-dependent calibration behaviour.

Mediated electron transfer is recognised by low-bias operation near the mediator’s formal potential, persistence of the signal under nitrogen or low-oxygen conditions, and changes in sensitivity when mediator loading or film hydration is altered. Redox couples often appear in voltammetry when mediators are mobile or partially leached. Diagnostic controls include air vs. nitrogen, mediator removal or poisoning, stoichiometry determinations under controlled O_2_ and explicit leaching tests.

Bienzymatic ChOx-HRP stacks detect H_2_O_2_ at low bias through the HRP-mediator cycle, in which each peroxide molecule oxidises two mediator equivalents when one-electron mediators are used. Oxygen has little influence once H_2_O_2_ reaches HRP, and Michaelis-Menten behaviour often emerges at low potential. Controls include catalase addition, omission or replacement of HRP, and bias stepping around the mediator potential to confirm that the signal follows the HRP mediator rather than peroxide oxidation.

Claims of DET require exceptional scrutiny because the flavin cofactor is buried and structurally inaccessible to the electrode. Apparent DET commonly arises from redox-active supports or electrocatalytic films. Hallmarks include responses that persist after enzyme denaturation, measurable activity in enzyme-free controls and voltammetric features that can be assigned to the support rather than to enzymatic turnover. Robust testing, therefore, demands heat denaturation or proteolysis, film-only electrodes and oxygen-dependence studies.

Common mechanistic pitfalls can be identified experimentally. Oxygen-limited turnover produces calibration slopes that collapse at low O_2_ and a stoichiometry that trends toward one H_2_O_2_ per choline. Mediator competition with oxygen can make mediated systems appear oxygen-dependent, while mediator leaching causes drifting baselines and loss of activity after rinsing or repeated cycling. These behaviours are diagnostic of transport and stability problems rather than of the intrinsic enzyme kinetics.

Meaningful comparison of calibration slopes and sensitivity requires matched O_2_ conditions, known film thickness and hydration, mediator content, reference electrode identity and mechanism-specific controls. Table 4 summarises the key mechanistic distinctions that determine bias windows, selectivity and operational potential across architectures.

Mechanistic interpretation is therefore central to evaluating choline sensors. Correctly identifying the electron-transfer route, whether peroxide-based, mediated, HRP-mediated or artefactual, enables reliable comparison across device formats and underpins the reporting standards required for reproducibility and translation.

## 6. Meta-Analysis and Design Trade-Offs

Having defined diagnostic criteria for interpreting electron transfer pathways, we now examine how these mechanistic constraints shape analytical performance through different platforms. Across the literature compiled in Table 2 and Table 3, three variables dominate analytical performance: the applied potential, the transport architecture of the sensing layer and the strategy used to regenerate the redox centre. These factors determine selectivity, speed and stability and account for many of the apparent discrepancies between studies.

The first-generation peroxide mode requires a positive bias and therefore promotes the oxidation of ascorbate, urate, and paracetamol; selectivity then rests on permselective and anti-fouling barriers, as well as self-referencing geometries [17,19,32]. Moving the readout window toward zero with PB, or to negative operation with bienzymatic ChOx-HRP systems, reduces these interferents but introduces dependence on catalyst or mediator integrity [46,50,53]. Mediated electron transfer can operate from −0.2 to +0.5 V when the relay is well positioned, improving selectivity relative to peroxide mode while avoiding the constraints associated with strongly negative bias [59,60,64]. In practice, the potential window best suited to the matrix should be identified first, followed by the chemistry that can operate reliably within that window.

Thin, hydrated and lightly cross-linked films minimise transport distances and shorten response time but increase the risk of mediator or enzyme loss. Thicker or more cross-linked matrices dampen transients and can obscure turnover changes caused by O_2_ availability or hydration state [37,40]. In peroxide mode, these structural effects often appear as deviations from linearity near the upper end of the calibration; in mediated architectures, they influence the extent to which the mediator effectively competes with solution-phase oxidants [17,18].

Covalent attachment and redox hydrogels reduce leaching and extend device lifetime but can increase tunnelling distance or reduce local water content, slowing electron transfer [62,63]. Physical entrapment and nanocarbon adsorption provide rapid responses but often require periodic regeneration or disposable formats [43,53]. In bienzymatic stacks, the mediator coupling HRP to the electrode is frequently the determinant of operational stability: osmium or ferrocene mediators in polymer backbones provide the most durable performance, whereas small-molecule mediators trade longevity for ease of renewal in flow-through or co-facing geometries [31,76,77,78,102].

Large-area screen-printed platforms deliver low noise and support long-term operation but are more susceptible to gradual baseline drift; they are suitable for high-throughput serum and food analysis where frequent blanking is feasible [50,73]. Microelectrodes with enzyme coatings provide short diffusion paths, rapid mass transport and low capacitance, sharpening responses and improving detection limits in brain extracellular space and microdialysis streams, but require careful matching of permselective barriers in self-referencing arrays [17,19,32]. Microdialysis decouples sampling from the detector and should always report in situ recovery to enable comparison across probes and flow rates [17,25].

In calibration-free and ratiometric strategies, coulometric dosing and signal normalisation improve robustness but rely on an assumed electron count. The operative *n* should be verified under both air and nitrogen, with catalase or mediator-removal controls included before fixing *n* in a model [31,34,35]. Self-referencing reduces drift only when the reference and sensing channels match in barrier chemistry, thickness and area; even minor mismatches introduce curvature that leaks into the differential trace [19,32].

Relay potential and size must suit the buried and largely apolar access path to the flavin. Compact neutral relays, such as benzoquinone and immobilised ferrocene, consistently perform well, whereas highly charged species are slowed by desolvation penalties and interactions with film electrostatics and tend to report film permeability rather than genuine side transfer [60,61,66,68]. In bienzymatic stacks, the HRP mediator must be fast and reversible under the operating conditions and compatible with the ionic composition of the matrix [76,77,78,82]. For PB, the stability of the mixed-valence lattice and the chloride content govern drift and pH tolerance and should be matched to the intended matrix [50].

### 6.1. Comparative Performance Across Architectures

Across the data in Table 2 and Table 3, no single architecture delivers uniformly superior performance; the preferred platform depends on the required potential window, matrix complexity and acceptable oxygen dependence. First-generation peroxide mode offers large signal amplitudes and wide linear ranges, but only PB-modified transducers operating near 0.0 V provide adequate selectivity in complex matrices while retaining complete oxygen dependence.

Second-generation mediated electron transfer offers oxygen-independent turnover when compact, neutrally charged mediators with well-placed potentials are used. Immobilised benzoquinone and ferrocene consistently deliver low-bias, low-noise operation in thin hydrated films. Diffusional benzoquinone is chemically fragile and unsuitable for complex matrices, whereas membrane-entrapped or scaffold-bound forms maintain predictable behaviour.

Bienzymatic ChOx-HRP stacks reliably deliver the lowest operational potentials (0.0 to –0.3 V) and the most potent suppression of interferents in serum, milk and brain microdialysate. Their long-term stability depends on mediator integrity and on maintaining HRP in kinetic excess. Ion-selective electrodes provide the only mature neutral-pH non-enzymatic option and offer sub-micromolar detection limits with excellent drift resistance. However, their selectivity against structurally similar quaternary ammonium species limits their use in some biological matrices.

Overall, apparent sensitivities can be high across all three enzymatic classes, depending on the choice of mediator or catalyst. Still, low-potential PB platforms and bienzymatic stacks consistently provide the most robust performance in interferent-rich samples. Architecture selection, therefore, depends less on maximum sensitivity and more on matching the potential window and transport constraints to the matrix, with oxygen-independent mediated transfer and bienzymatic HRP wiring offering the most reliable operation when low bias and matrix compatibility are prioritised.

### 6.2. Outstanding Challenges and Future Directions

Despite substantial progress across peroxide-based, mediated, and bienzymatic architectures, several challenges continue to limit reproducibility and long-term deployment. Chief among these are the stability of immobilised enzymes and mediators, the sensitivity of signal generation to oxygen transport and hydration state, and the difficulty of maintaining predictable behaviour in fouling-prone biological matrices. Many reported devices optimise sensitivity under idealised conditions, while comparatively fewer studies evaluate robustness under extended operation, temperature variation, or repeated exposure to real samples. In parallel, disposable and single-use formats have emerged as a practical deployment strategy, particularly when combined with low-potential PB or HRP-coupled readouts, enabling sensitive point-of-care measurements without requiring long operational lifetimes.

From a design perspective, future advances are likely to arise from architectures that decouple signal generation from oxygen availability, improve the reversibility and retention of redox mediators, and reduce dependence on tightly controlled film microenvironments. Low-potential operation remains a unifying requirement for translation, favouring mediated electron transfer and bienzymatic formats when long-term stability can be ensured. However, achieving this reliability requires careful balancing of mediator chemistry, immobilisation strategy, and film transport properties, rather than incremental gains in analytical sensitivity alone.

More broadly, progress in electrochemical choline sensing will depend on tighter integration between mechanistic characterisation, materials design, and validation protocols. Systematic reporting of electron transfer pathways, mediator cycling behaviour, and matrix-dependent performance will be essential for resolving apparent discrepancies across the literature. Addressing these challenges will be critical for advancing electrochemical choline sensors beyond proof-of-concept demonstrations and toward platforms capable of predictable, application-relevant performance.

## 7. Translational Use Cases

The design choices outlined above translate differently across biological matrices, where concentration ranges, interferent profiles and allowable bias windows vary sharply. Because these constraints arise from the matrix rather than the species, the chemical environment primarily sets the required specifications, and most device studies are conducted in human samples unless otherwise noted. Representative device performance across key matrices is summarised in Table 2 and Table 3.

In the brain extracellular space, choline typically sits in the low micromolar range (Table 1) and varies with cholinergic tone and transporter activity. Ceramic or carbon microelectrode arrays with paired choline and sentinel channels have captured second-scale fluctuations in vivo while suppressing motion artefacts and interferents [17,19,32]. In this matrix, realistic analytical targets are LoD ≤ 1 µM, a linear range spanning at least 1–50 µM, second-scale response times, and operation at potentials that minimise ascorbate oxidation and co-oxidation of catecholamines. First-generation peroxide readout can meet the response-time requirement when thin poly(m-PD)/Nafion films are used, although the positive bias necessitates strict self-referencing. Low-bias MET (0.0 to about +0.2 V) reduces interferent pressure, provided oxygen independence is demonstrated through air-nitrogen and catalase controls. A practical configuration is a matched-barrier microarray operated either in peroxide mode near 0.0 V vs. Ag/AgCl, 3 M NaCl or with immobilised ferrocene at low positive bias, with explicit reporting of reference potentials and in situ recovery when microdialysis is used upstream [17,19,32,50,59].

In clinical serum and whole blood, adult plasma choline levels are in the high single-digit micromolar range, with broad inter-individual variation, particularly in pregnancy, liver disease, and metabolic stress [1,3,10,103]. The interferent burden is substantial because ascorbate, urate, paracetamol and proteins are abundant. Target specifications include LoD ≤ 1 µM, a linear range extending to ≥200 µM to accommodate supplementation and outliers, and, where possible, operation at or below 0.0 V to suppress matrix oxidation. Disposable screen-printed electrodes are well-suited when the chemistry matches the matrix window: Prussian Blue supports near-zero-potential peroxide detection with good interferent tolerance, while bienzymatic ChOx-HRP allows zero- or negative-potential operation by consuming peroxide within the stack. Low-bias MET remains viable when barrier stacks are rigorous and oxygen controls are performed. Brief dilution and filtration reduce fouling without compromising the diagnostic range. A practical point-of-care choice is a screen-printed working electrode with PB or a wired peroxidase mediator, a short Nafion layer and a stable reference system [51,73,76,78].

In human milk across lactation, free choline typically sits in the tens of micromolar, while phosphocholine and glycerophosphocholine contribute to a larger total pool that varies across colostrum, transitional and mature milk [11,12]. The high fat and protein content demands disposable detectors and robust anti-fouling strategies. Target specifications include an LoD of 1–5 µM for free choline and a linear range extending to the millimolar level for total choline or for measurements following enzymatic hydrolysis of conjugated forms. Architectures operating at or below 0.0 V (e.g., PB, bienzymatic wiring, or suitably tuned low-bias MET) deliver the most reliable selectivity once a defined dilution and phase-separation step is applied [11,12,50,78].

Within one-carbon metabolism and nutritional monitoring, plasma choline and its metabolites serve as indicators of intake, methyl-donor status and liver function [1,3,4,103]. The matrix is again serum or plasma, so the same analytical targets apply, but long-term stability, calibration practice and inter-laboratory comparability become dominant concerns. Low-bias designs with stable referencing support reproducible longitudinal data. A practical choice for extended studies is a benchtop flow cell with a co-facing geometry, using enzymes in solution and a reversible mediator at near-zero potential, which simplifies charge accounting and blanking between runs. Where portability is needed, PB-based or bienzymatic screen-printed platforms remain suitable provided storage conditions, electrode conditioning and matrix-matched calibration are controlled [31,50,76,78].

From a translational and commercial perspective, most electrochemical choline sensors remain at the proof-of-concept stage, with deployment limited by enzyme lifetime, mediator stability, and the need for matrix-specific calibration. Devices intended for real-world use must prioritise robustness, simplicity, and compatibility with disposable manufacturing formats over maximal sensitivity. In practice, point-of-care translation is most feasible for low-bias architectures that tolerate short operational lifetimes, particularly screen-printed platforms incorporating PB or bienzymatic ChOx-HRP stacks. By contrast, continuous or long-term monitoring applications impose stricter requirements on stability, referencing, and oxygen independence, which are currently met only in specialised laboratory configurations. Bridging this gap will require standardised validation in real samples, explicit reporting of operational lifetimes, and performance benchmarking under clinically or environmentally relevant conditions rather than idealised buffers.

Across translational settings, the architecture that delivers the most robust performance is the one that aligns its operating potential with the local interferent landscape while maintaining controlled mass transport. In plasma and milk, PB and bienzymatic stacks commonly offer the most reliable low-bias operation; in the brain extracellular space and dialysate sampling, microelectrodes using stable mediated electron transfer provide rapid, reproducible readouts. When matrix constraints, bias limits and transport geometry are matched to the sensing chemistry, the choice between peroxide mode, mediated transfer and bienzymatic stacks can be made on practical grounds rather than on conflicting literature claims.

## 8. Conclusions

Electrochemical choline sensing has advanced substantially over the past decades, but genuine translation still hinges on understanding how biology, electron transfer pathways and reporting practice intersect. Framing choline within its physiologically relevant ranges and matrix-specific constraints makes clear that successful devices must be designed around the realities of plasma, milk, cerebrospinal fluid and the brain extracellular space rather than idealised buffer conditions. Mechanistic clarity is central to this process: distinguishing peroxide-based readouts from mediated electron transfer and from the far rarer and often misassigned direct electron transfer pathways prevents misinterpretation of sensitivity, stoichiometry, and stability.

Across architectures, low-bias mediated systems and well-controlled bienzymatic stacks remain the most robust routes to oxygen-independent operation. At the same time, DET claims for FAD enzymes continue to require stringent controls before they can be considered viable for field or clinical deployment. Non-enzymatic and transistor-coupled platforms offer promising forward pathways, particularly where neutral-pH operation, anti-fouling performance or integration with wearable and implantable formats is required. Still, these technologies will need rigorous benchmarking against enzymatic baselines before adoption.

These gaps mirror the mechanistic ambiguities highlighted throughout Section 3, Section 4, Section 5, Section 6 and Section 7 and emphasise that reported device performance cannot be disentangled from the underpinning electron transfer pathway. Despite substantial progress, several gaps continue to limit comparability and translation. Long-term stability assessments are sparse and rarely performed in real matrices; oxygen dependence is inconsistently characterised; and many studies do not provide sufficient detail about the reference electrode, such as the chloride concentration or type of Ag/AgCl system, to allow reliable conversion to a standard reference scale, which obstructs meaningful evaluation of bias windows. Mediator integrity is often inferred rather than measured directly, and the stability of PB lattices, chloride sensitivity and the requirement for HRP excess in bienzymatic stacks remain underexplored. Matrix-matched calibration and reporting of microdialysis recovery are still exceptions rather than standard practice. Finally, claims of DET frequently lack the mechanistic controls needed to substantiate them, impeding clear interpretation across platforms.

Looking ahead, the field is well-placed to deliver reliable, application-ready choline sensors. Achieving this will depend on coupling mechanistic insight with practical validation: oxygen-controlled experiments, matrix-matched calibration, drift quantification and head-to-head comparison with established methods such as LC-MS/MS. With these elements in place, choline sensing can move beyond proof-of-concept electrodes toward reproducible, deployable tools that support neuroscience, nutrition, clinical diagnostics and agricultural monitoring.

## Figures and Tables

**Table 1:** Representative concentrations of choline across species and matrices.

Species	Matrix	Typical Concentration (µM) ^1^	Notes	Ref.
Human	plasma	8–20	Fasting adults; rises during pregnancy due to increased phosphatidylcholine turnover	[1,2,3,4,10]
	milk	700–1400	Total choline expressed as free-choline equivalents; water-soluble forms comprise about eighty to ninety percent and vary with lactation stage	[11,12]
	CSF	1–3	Lumbar CSF in healthy adults; HPLC and enzymatic studies report about two micromolar with age-related variation	[13,14,15]
Rat	plasma	11	Untreated Wistar rats	[16]
	CSF	7		
	ECS	3–12	Basal cortex extracellular space measured in vivo; varies with brain region, probe design and anaesthesia	[17,18,19]
Bovine	plasma	8–16	Diet and lactation stage dependent; relevant for metabolic and nutritional studies	[20,21,22]
	milk	500–900	Total choline (sum of free, glycerophosphocholine, and phosphatidylcholine); diet-dependent composition	
Canine	plasma	6–10	Healthy adult dogs. It increases to ca. 15 µM when fed commercial diets	[23,24]

^1^ “Typical concentration” refers to free choline unless “total” is specified in the notes section.

**Table 2:** Representative first-generation choline sensors across matrices, electrode systems, applied potentials, and key features.

Sample/Matrix	Electrode/System	Potential (V vs. Ag/AgCl, 3 M NaCl) ^1^	Linear Range (µM)	LoD (µM)	Additional Info	Ref.
Rat frontal cortex (in vivo)	4-channel ceramic MEA; ChOx vs. sentinel	+0.7	0.7–80	0.2	Self-referencing subtraction; improved selectivity	[33]
0.1 M phosphate buffer pH 7	Pt/ChOx	+0.7	0.7–1000	0.7	Enhanced H_2_O_2_ oxidation; moderate interferent control	[37]
0.1 M phosphate buffer pH 7.4	ChOx-EACC-PB/Pt	0.0	0.5–100	0.5	Low-potential PB transducer; fast response and high sensitivity	[45]
	ChOx-PB-BL WCNT-IL/GC	−0.05	0.5–100	0.5	Fast response; high stability; interferent rejection	[46]
	ChOx-MWCNT/Pt	+0.17	5–100	0.1	CNT electrocatalysis lowers working bias and improves selectivity	[47]
0.1 M phosphate buffer pH 8	ChOx-PVA-AuNr/Pt	+0.4	20–400	10	Au nanorods markedly enhance conductivity and current response	[48]
0.2 M PBS pH 7.0	ChOx/IL-NH_2_-MWCNT/GC	−0.45	5–800	3.9	Hydrophilic ILs give highest stability, sensitivity and widest range	[49]
0.1 M PBS pH 7.4	ChOx-PB/SPCE	−0.05 ^2^	0.5–100	0.5	Long-term stability; low-potential detection	[50]
0.1 M PBS pH 7.6	ChOx/MWCNT-AuNP-PDDA/Pt	+0.36	1–500	0.3	CNT-AuNP synergy boosts sensitivity and lowers working bias	[51]
0.1 M PBS pH 7.8	ChOx/ZnO-MWCNT-PDDA/PG	+0.6	1–800	0.3	Synergistic ZnO-CNT film gives high sensitivity and long-term stability	[52]
0.1 M PBS pH 8	ChOx-PDDA-PB-FePO_4_/GC	−0.08	2–3000	0.4	PB low-potential H_2_O_2_ reduction; strong interferent rejection	[53]
0.1 M PBS pH 8.5	ChOx/AuNP/MWCNT/GCE	−0.3	3.3–120	0.6	Mixed CNT-AuNP layer gives high sensitivity and improved one-month stability	[54]

^1^ All potentials are reported vs. Ag/AgCl, 3 M NaCl. Potential values reported vs. alternative reference electrodes were converted using Ag/AgCl, 3 M NaCl, and Ag/AgCl, sat. KCl = −0.034, −0.042 V vs. SCE, respectively [55,56,57]. ^2^ Potential reported vs. internal screen-printed Ag pseudo-reference electrode.

**Table 3:** Representative bienzymatic ChOx plus HRP choline sensors.

Sample or Matrix	Electrode or System	Applied Potential (V vs. Ag/AgCl, 3 M NaCl) ^1^	Linear Range (µM)	LoD (µM)	Mediator Between HRP and Electrode	Notes	Ref
PBS pH 7.4	ChOx-HRP-PVP-Os(bpy)_2_ Cl/GC	−0.13	1–100	–	PVP-Os(bpy)_2_Cl	Classic wired HRP stack operated at low bias with fast response of about 2 s	[81]
0.1 M PBS pH 7.4	ChOx-HRP-Fc-CRGO/GC	−0.13	1–400	0.4	Ferrocene-CRGO nanohybrid	Fast, low-bias response; 95% signal in 8 s	[59]
1/15 M phosphate buffer pH 7.4	ChOx-HRP-PTH/CPE	−0.23	5–600	3	PTH redox film	PTH shows efficient electron transfer with HRP at negative bias; CPE reproducibility issues	[82]
0.1 M phosphate buffer pH 7.4	ChOx-HRP-poly-TTCA/GC	−0.19	1–80	0.1	Conducting poly-TTCA film	Cathodic detection of peroxide on poly-TTCA supports low-potential operation	[76]
	ChOx-HRP/CPE	0	0.05–70	0.01	Phenothiazine	Low-bias reduction; stable mediated HRP transduction; CPE reproducibility issues	[83]
0.1 M Tris buffer pH 8	ChOx-HRP-PDDA-MWCNT/GC	−0.20	50–5000	10	None added, CNT surface provides electrocatalysis and possible direct wiring of HRP	Authors note possible direct reduction of peroxide at CNT at low potential	[78]
0.1 M phosphate buffer pH 7.5	ChOx-HRP- PVI_13_-dmeOs/CPE	−0.13	1–10	0.1	PVI_13_-dmeOs redox polymer	Early example of low-potential wired HRP for peroxide reduction in choline sensors; CPE reproducibility issues	[74]
infant formula	HRP-ChOx-CNF MnO_2_-PMG/SPCE	−0.20 ^2^	4–9000	0.8	PMG	Improved stability and low-bias H_2_O_2_ transduction	[73]

^1^ All potentials are reported vs. Ag/AgCl, 3 M NaCl. Potential values reported vs. alternative reference electrodes were converted using Ag/AgCl, 3 M NaCl; Ag/AgCl, 0.1 M KCl; and Ag/AgCl, sat. KCl = −0.034, 0.047, and −0.042 V vs. SCE, respectively [55,56,57]. ^2^ Potential reported vs. internal screen-printed Ag pseudo-reference electrode.

**Table 4:** Mechanistic comparison of choline sensor architectures.

Feature	First Generation (H_2_O_2_ Readout)	MET	Bienzymatic (ChOx-HRP)	Purported DET
Terminal oxidant	O_2_	Mediator	Mediator via HRP	Electrode (claimed)
Operating potential	High positive	Low bias (mediator-defined)	Low bias	Variable, typically positive
O_2_ dependence	Strong	Present unless mediator dominates	Strong (ChOx-limited)	None (if genuine)
Stoichiometry	1–2 H_2_O_2_ per choline	2–4 e^−^ depending on mediation	2 mediator equivalents per H_2_O_2_ *	Unverified
Failure modes	Interferents, O_2_ limitation	Mediator leaching, incomplete mediation	HRP instability	Redox-film artefacts
Matrix compatibility	Moderate-low	Moderate-high	High	Unverified

* Considering a one-electron mediator.

## Data Availability

No new data were created or analysed in this study. Data sharing does not apply to this article.

## Abbreviations

The following abbreviations are used in this manuscript:

ACh	acetylcholine
AuNP	gold nanoparticles
AuNr	gold nanorods
BL-MWCNT	bamboo-like multi-walled carbon nanotube
bpy	2,2′-bipyridine
CB	carbon black
ChOx	choline oxidase
CPE	carbon paste electrode
CNF	carbon nanofibres
CNF-MnO_2_	MnO_2_ nanoparticles decorated carbon nanofibres
CNT	carbon nanotube
CPME	poly-5,2′:5′,2″-terthiophene-3′-carboxylic acid modified electrode
CRGO	chemically reduced graphene oxide
CSF	cerebrospinal fluid
CSPE	carbon screen-printed electrode
DET	direct electron transfer
EACC	6-*O*-ethoxytrimethylammoniochitosan chloride
ECS	extracellular space
GC	glassy carbon
GNP	gold nanoparticles
GPC	glycerophosphocholine
HRP	horseradish peroxidase
HPLC	high-performance liquid chromatography
IL	ionic liquid
ISE	ion-selective electrode
LC	liquid chromatography
LC-MS/MS	liquid chromatography-tandem mass spectrometry
LoD	limit of detection
MB	meldola blue
MEA	microelectrode array
MET	mediated electron transfer
m-PD	meta-phenylenediamine
MWCNT	multi-walled carbon nanotube
NH_2_-MWCNTs	amine-functionalised multi-walled carbon nanotubes
NWs	nanowires
PANI	polyaniline
PB	Prussian blue
PBS	phosphate-buffered saline
PC	phosphatidylcholine
PDDA	poly(diallyldimethylammonium chloride)
PMG	poly(methylene green)
PPy	polypyrrole
Pt	platinum
PTH	poly(thionine)
PVA	polyvinyl alcohol
PVP	poly(4-vinylpyridine)
PVS	polyvinylsulphonate
SBA-15	mesoporous silica powder
SCE	saturated calomel electrode
SHE	standard hydrogen electrode
SPCE	screen-printed carbon electrode
TBO	toluidine blue O
TD-p-AgSA	tubular detector of polished silver solid amalgam
TTCA	5,2′:5′,2″-terthiophene-3′-carboxylic acid
